# Supplementary material for: In Vitro and In Vivo Characterisation of a Mucoadhesive Buccal Film Loaded with Doxycycline Hyclate for Topical Application in Periodontitis
Source: Pharmaceutics. 2023 Feb 8;15(2):580. doi: 10.3390/pharmaceutics15020580 (PMC9963859; doi:10.3390/pharmaceutics15020580)
Supplement: Supplementary file 1 [file pharmaceutics-15-00580-s001.zip › pharmaceutics-2196915-supplementary.pdf]

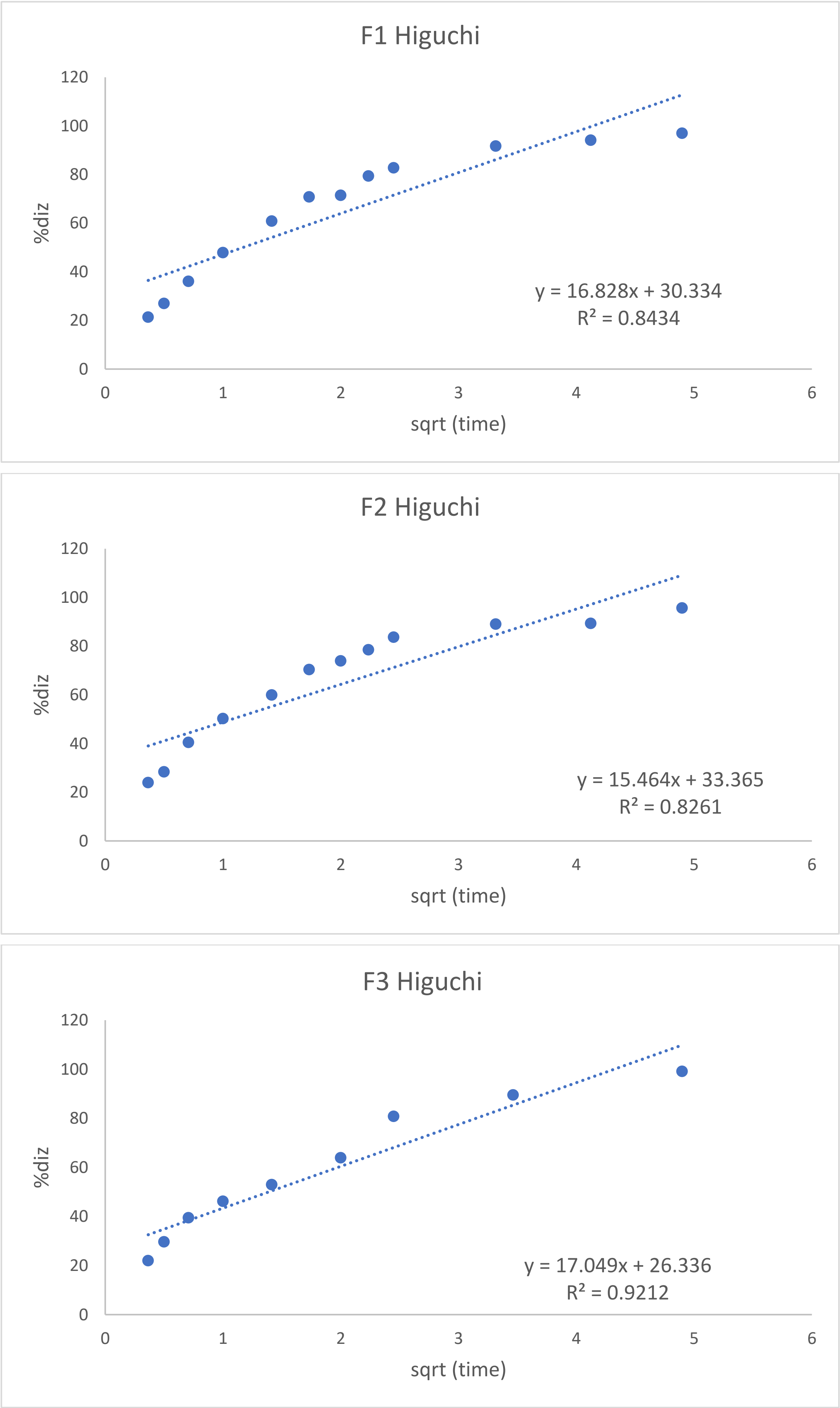
F1 Higuchi
%diz
sqrt (time)
y = 16.828x + 30.334
R² = 0.8434
F2 Higuchi
%diz
sqrt (time)
y = 15.464x + 33.365
R² = 0.8261
F3 Higuchi
%diz
sqrt (time)
y = 17.049x + 26.336
R² = 0.9212

(a)

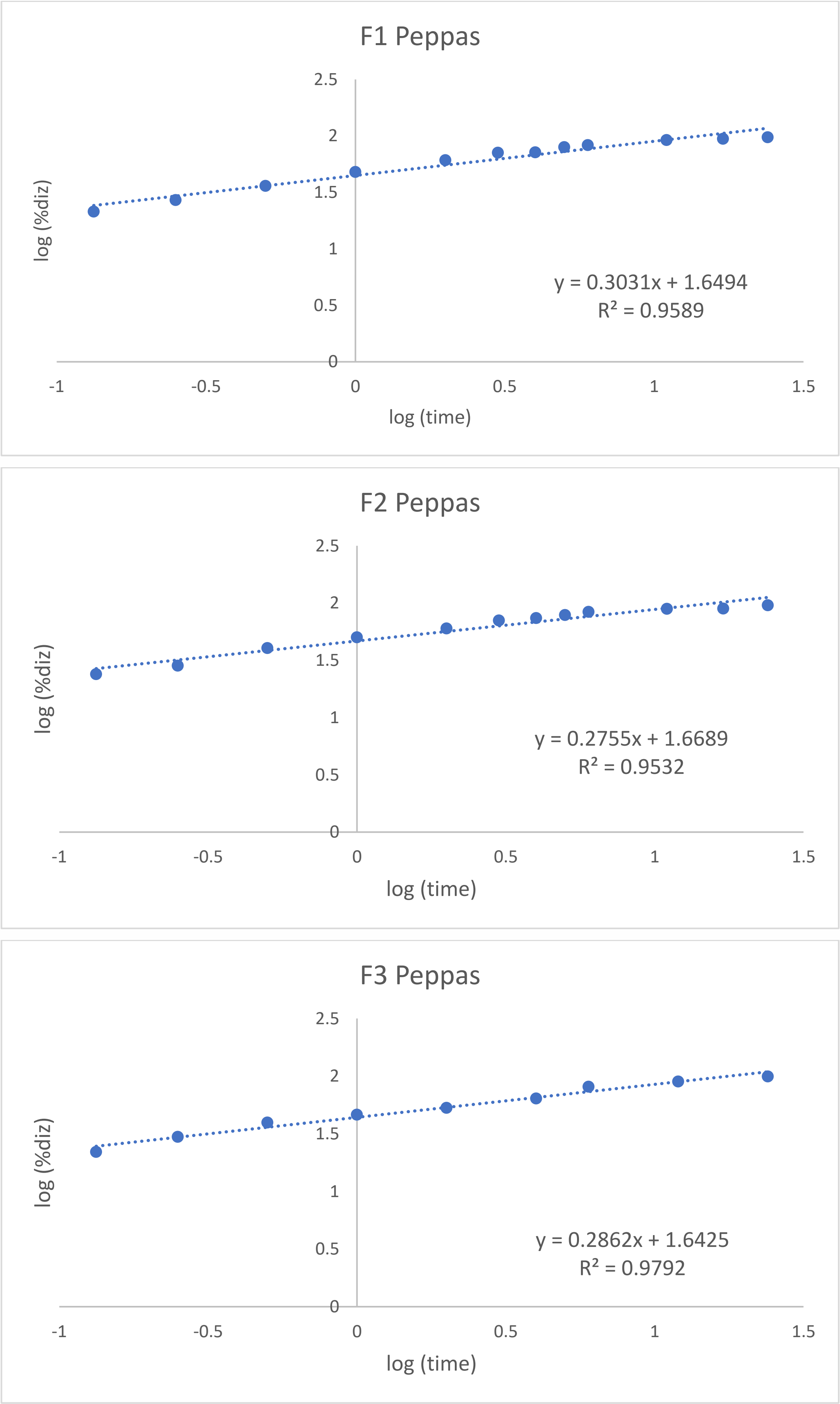

(**b**)

Figure S1. (**a**) Fitting the realease data to the Higuchi mathematical model (F1–F3, according to Table 1). (**b**) Fitting the realease data to the Peppas mathematical model (F1–F3, according to Table 1).

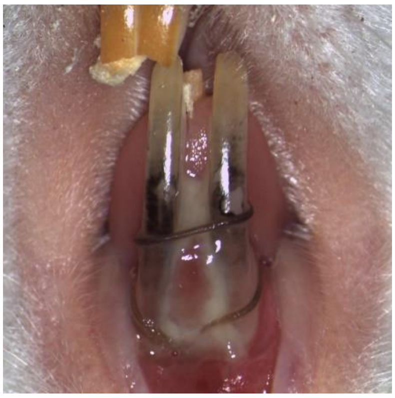

Figure S2. Clinical aspect of the ligatures on the lower incisors, 14 days after application (10x magnification).

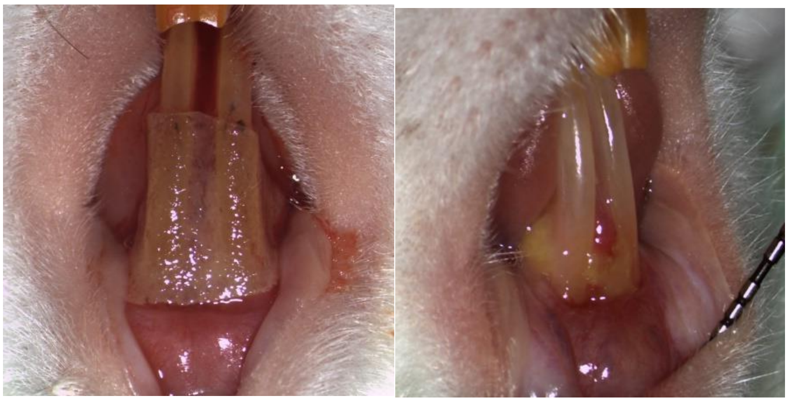

(**A**) (**B**)

Figure S3. Clinical aspect of the Doxy Hyc mucoadhesive buccal film applied on the gingiva (10x magnification): (**A**) after application; (**B**) after three days.
